# Supplementary material for: A coiled-coil-based design strategy for the thermostabilization of G-protein-coupled receptors
Source: Sci Rep. 2023 Jun 22;13:10159. doi: 10.1038/s41598-023-36855-1 (PMC10287670; doi:10.1038/s41598-023-36855-1)
Supplement: Supplementary file 1 — Supplementary Information. [file 41598_2023_36855_MOESM1_ESM.docx]

**SUPPLEMENTARY INFORMATION**

**A Coiled-Coil-Based Design Strategy for the Thermostabilization of G-Protein-Coupled Receptors**

Marwa Amer, Oneda Leka, Piotr Jasko, Daniel Frey, Xiaodan Li and Richard A. Kammerer*

Laboratory of Biomolecular Research, Division of Biology and Chemistry, Paul Scherrer Institute, 5232 Villigen PSI, Switzerland.

* Corresponding author: Richard A. Kammerer, Laboratory of Biomolecular Research, Division of Biology and Chemistry, Paul Scherrer Institute, 5232 Villigen PSI, Switzerland.

E-mail: richard.kammerer@psi.ch


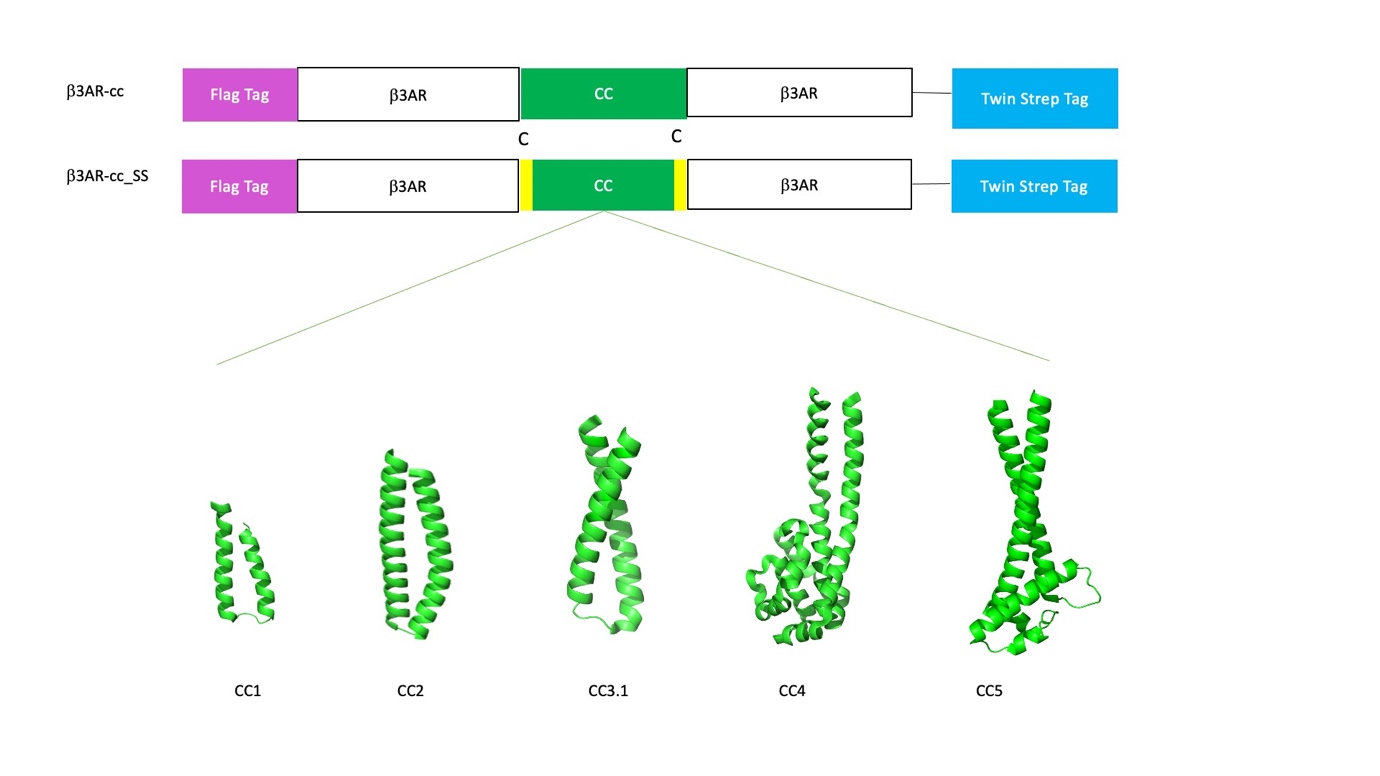


**Supplementary Figure S1.** **Design of β3AR-cc constructs.** Schematic representation of the chimeric β3AR variants and crystal structures of coiled-coil domains that were used for replacing ICL3 of β3AR. For cc3.2 there is no structure available. The constructs have a N-terminal Flag tag and C-terminal TwinStrep tag for western blot detection and protein purification.

**
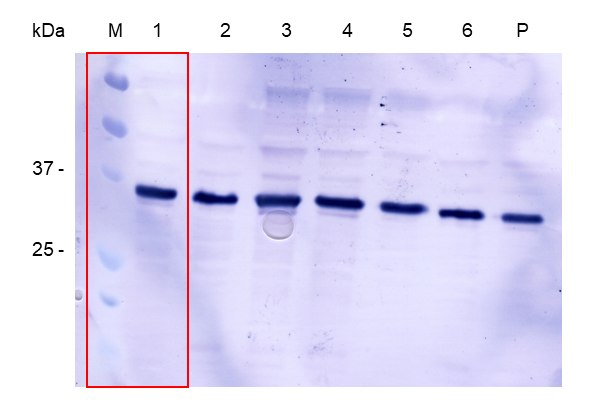
**

**Supplementary Figure S2. Unprocessed original image used for the generation of Figure 1d.** The part of the image that was used for the generation Figure 1d is surrounded by a red box.


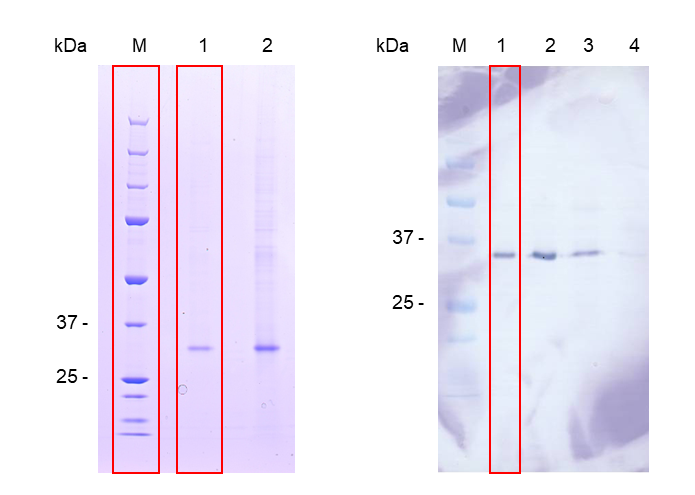


**Supplementary Figure S3. Unprocessed original images used for the generation of Figure 5a.** The parts of the images that were used for the generation Figure 5a are surrounded by a red box.

**
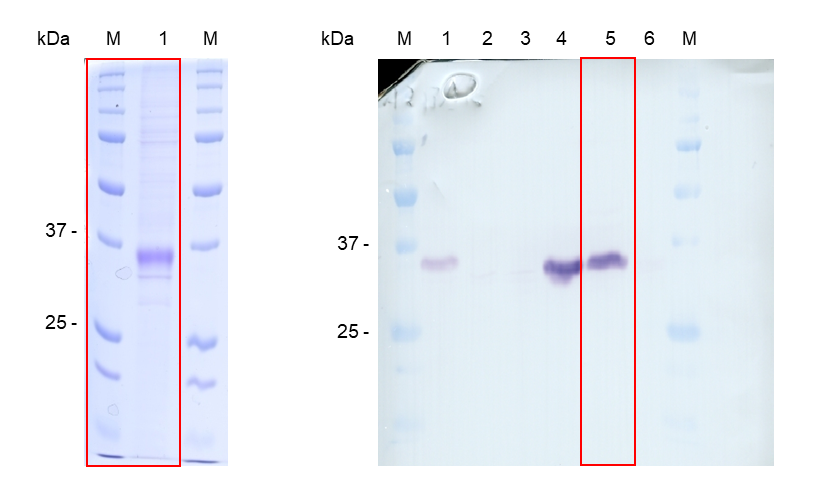
**

**Supplementary Figure S4. Unprocessed original images used for the generation of Figure 5b.** The parts of the images that were used for the generation Figure 5b are surrounded by a red box.


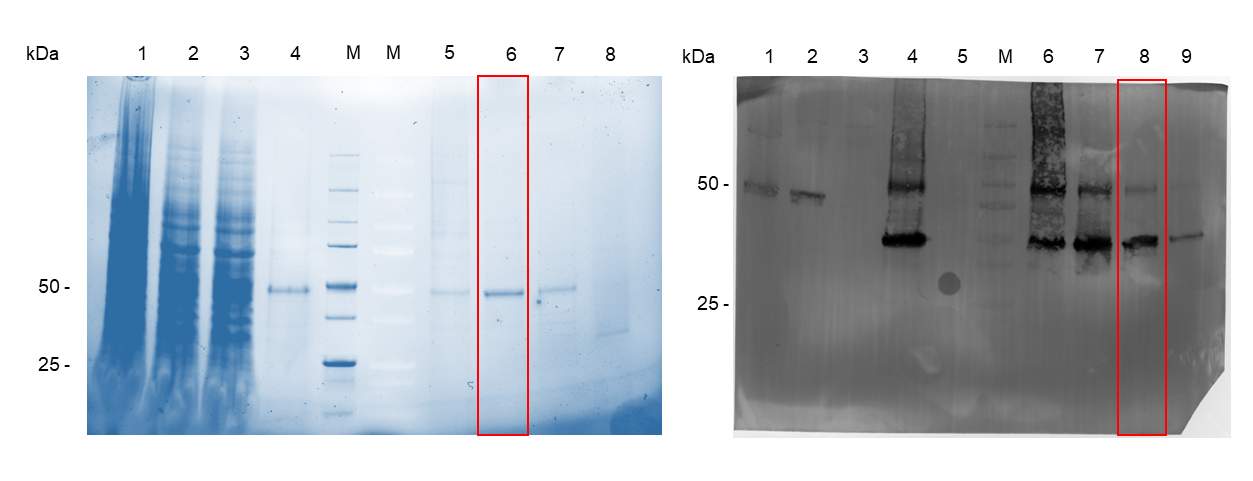


**Supplementary Figure S4. Unprocessed original images used for the generation of Figure 7c.** The parts of the images that were used for the generation Figure 7c are surrounded by a red box.

**Supplementary Table 1.**

| Coiled-coil | Sequence | PDB ID | Resolution |
| --- | --- | --- | --- |
| cc1 | CKIEHAKKKRLFDLYINGSYEVSELDSMMNDIDAQINYC | 5UDO | 2.5 Å |
| cc2 | LKEVQDNITLHEQRLVTTRQKLKDAERAVELDPDDVNKSTLQSRRAAVSALETKLGELKRELADL | 2IC6 | 1.15 Å |
| cc3.1 | LLALDREVQELKKRLQEVQTERNQVAKRVPKAPPEEKEALIARGKALGEEAKRLEEALREKEARLEAL | 1SRY | 2.5 Å |
| cc3.2 | VRELDELWRKKLQEVNEVRHKHNVVTRMIAKARDPEERKRLIEEARRLLKLREELEKELKRIEEEREKL | NA | NA |
| cc4 | LKEEQERKAEIQADIAQQEKNKAKLVVDRNKIIESQDVIRQYNLADMFKDYIPNISDLDKLDLANPKKELIKQAIKQGVEIAKKILGNISKGLKYIELADARAKLDERINQINKDCDDLKIQLKGVEQRIAGI | 6EK4 | 2.8 Å |
| cc5 | LLALLAVDEQLHKQQEVIADKQMSVKEDLDKVEPAVIEAQNAVKSIKKQHLVEVRSMANPPAAVKLALESIALLLGESTTDWKQIRSIIMRENFIPTIVNFSAEEISDAIREKMKKNYMSNPSYNYEIVNRASLAAGPMVKWAIAQLNYADMLKRVEPLRNELQKLEDDAKDNQQKLEAL | 3ERR | 2.2 Å |
| cc-T4L | LLALDREVQELKKRLQEVQTERNQVAKRVKRQLNIFEMLRIDEGLRLKIYKDTEGYYTIGIGHLLTKSPSLNAAKSELDKAIGRNTNGVITKDEAEKLFNQDVDAAVRGILRNAKLKPVYDSLDAVRRAALINMVFQMGETGVAGFTNSLRMLQQKRWDEAAVNLAKSRWYNQTPNRAKRVITTFRTGTWDAYKFCLKELIARGKALGEEAKRLEEALREKEARLEAL | NA | NA |

Amino acid sequences of the coiled-coil sequences used in this study. The underlined amino acids were changed to Cys in the disulfide bridge variants.
